# COVID‐19 and tobacco products use among US adults, 2021 National Health Interview Survey

**DOI:** 10.1002/hsr2.1542

**Published:** 2023-08-31

**Authors:** Mohammad Ebrahimi Kalan, Rime Jebai, Wei Li, Prem Gautam, Seyedeh Yasaman Alemohammad, Zeinab Mortazavizadeh, D. Ward Kenneth, Aditya Chakraborty, Ghader Dargahi Abbasabad, Raed Behaleh, Zoran Bursac, Ziyad Ben Taleb

**Affiliations:** ^1^ Eastern Virginia Medical School Norfolk Virginia USA; ^2^ Department of Epidemiology, Robert Stempel College of Public Health Florida International University Miami Florida USA; ^3^ Department of Psychiatry, Yale School of Medicine Yale University New Haven Connecticut USA; ^4^ Texas State Board of Pharmacy Austin Texas USA; ^5^ Department of Psychology The University of Alabama at Birmingham Birmingham Alabama USA; ^6^ School of Public Health University of Memphis Memphis Tennessee USA; ^7^ University of New Brunswick Fredericton California USA; ^8^ School of Health Sciences Baldwin Wallace University Berea Ohio USA; ^9^ Department of Biostatistics, Robert Stempel College of Public Health Florida International University Miami Florida USA; ^10^ Department of Kinesiology, College of Nursing and Health Innovation University of Texas at Arlington Arlington Texas USA

**Keywords:** adults, COVID‐19, smoking, tobacco use

## Abstract

**Background:**

Smoking and vaping are linked to lung inflammation and lowered immune response.

**Objective:**

Examine the prevalence of coronavirus disease 2019 (COVID‐19) cases, testing, symptoms, and vaccine uptake, and associations with tobacco product use.

**Methods:**

Data came from the 2021 National Health Interview Survey. The 2021 Sample Adult component included 29,482 participants with a response rate of 50.9%. We investigated COVID‐19‐related outcomes by tobacco product use status and reported national estimates. Multivariable regression models were performed accounting for demographics (e.g., age, sex, poverty level), serious psychological distress, disability, and chronic health conditions.

**Results:**

In our regression analyses, odds of self‐reported COVID‐19 infection were significantly lower for combustible tobacco product users (vs. nonusers; adjusted odds ratio [AOR = 0.73; 95% confidence interval [CI] = 0.62–0.85]). Combustible tobacco users also were less likely to report ever testing for COVID‐19 (AOR = 0.88; 95% CI = 0.79–0.98), ever testing positive for COVID‐19 (AOR = 0.66; 95% CI = 0.56–0.77), and ever receiving COVID‐19 vaccine (AOR = 0.58; 95% CI = 0.51–0.66) compared with their nonuser peers. Compared to nonusers, users of any type of tobacco who contracted COVID‐19 had higher odds of losing smell (AOR = 1.36; 95%CI = 1.04–1.77), which was more pronounced among exclusive e‐cigarette users. The odds of receiving vaccine were lower for all current exclusive tobacco product users compared to nonusers (AORs = 0.40 to 0.70).

**Conclusions:**

Continued monitoring of tobacco product use and its association with respiratory diseases such as COVID‐19 is crucial to inform public health policies and programs. In addition, efforts to promote vaccination, especially among tobacco product users, are warranted.

## Key points

*What's known*: Smoking and vaping are linked to lung inflammation and can lower immune response. *What's new*: Combustible tobacco users had lower odds of self‐reported coronavirus disease 2019 (COVID‐19) infection, testing, and vaccination compared with nonusers. Tobacco users who got COVID‐19 had higher odds of losing smell, especially exclusive e‐cigarette users. All current exclusive tobacco product users were less likely to get vaccinated compared with nonusers. *Clinical implications*: Continued monitoring of tobacco use and COVID‐19 is needed to inform public health policies. Results highlight the importance of promoting COVID‐19 vaccination among tobacco users. Quitting smoking and vaping could reduce COVID‐19 risks. Findings suggest tobacco use is linked to lower testing and vaccine uptake as well as higher olfactory symptoms if infected.

## 1 INTRODUCTION

Smoking remains the main cause of premature death in the United States (US) and globally.^1^, ^2^ Smoking and vaping are linked to lung inflammation and lowered immune response.^3^, ^4^ Previous research demonstrated that smoking increases the risk and severity of multiple respiratory diseases, including chronic obstructive pulmonary disease (COPD) and lung cancer.^5^ Remarkably, prior studies on the impact of smoking on the clinical severity of coronavirus disease 2019 (COVID‐19) have shown conflicting results. In the early COVID‐19 pandemic, some claimed protective effects of nicotine (mainly biological aspects on cell receptors) against COVID‐19 infection^6^, ^7^, ^8^; however, recent evidence suggests that nicotine (from any tobacco products) does not protect against COVID‐19.^4^, ^9^, ^10^ The current evidence suggests that conventional cigarette smoking is associated with a greater risk of severe COVID‐19 symptoms, as evidenced by the need for hospitalization.^4^

The adverse effects of smoking on vulnerability to respiratory infections are well‐established.^11^, ^12^ Nevertheless, the lack of association between current smoking and COVID‐19 outcomes in some studies^13^, ^14^, ^15^, ^16^, ^17^, ^18^ may not be surprising given that the effects of testing and vaccination for COVID‐19 among tobacco users have been understudied using large, nationally representative samples. In fact, current evidence on the association between smoking and COVID‐19 risk is inadequate and conflicting, highlighting the need for consistent national studies with rigorous study designs.^18^ A cohort study^13^ of vaccination against COVID indicated that vaccination was protective against death, with emphasis on current and former smokers relative to never smokers. Although evidence shows a lessened serological response to COVID‐19 vaccines in smokers,^19^ among vaccinated patients, current smokers had the lowest mortality rate, and former smokers and never‐smokers had comparable rates.^13^ Therefore, monitoring the frequency of receiving vaccines (though cross‐sectionally) along with other characteristics of COVID‐19 (e.g., symptoms) might be useful for addressing vaccine hesitancy in these groups.^20^ and informing tobacco users about the positive consequences of vaccines. In this study, we analyzed data from the 2021 National Health Interview Survey (NHIS) to examine the prevalence of COVID‐19, testing, symptoms, and vaccine uptake among users of different tobacco products among a nationally representative sample of US adults aged ≥18 years.

## 2 METHODS

### 2.1 Study design and participants

NHIS is an annual, nationally representative survey of the noninstitutionalized US civilian population.^21^ The 2021 Sample Adult component included 29,482 adults aged ≥18 with a response rate of 50.9%.^21^ The Sample Adult module included specific questions about COVID‐19.^21^ This study followed the Strengthening the Reporting of Observational Studies in Epidemiology (STROBE) reporting guideline.

The survey description and data collection methodology for the 2021 NHIS can be found at the Centers for Disease Control and Prevention website at https://ftp.cdc.gov/pub/Health_Statistics/NCHS/Dataset_Documentation/NHIS/2021/srvydesc-508.pdf. In brief, due to the COVID‐19 pandemic, typical data collection procedures were disrupted in 2021 NHIS. From January to April, household members were initially contacted via telephone, with subsequent personal visits authorized. This approach continued from the latter part of 2020. Starting in May 2021, interviewers returned to standard survey interviewing procedures, where initial contact with household members was attempted in person, followed by telephone if necessary. Interviewers were required to wear masks, practice social distancing, and be fully vaccinated if interviews were conducted in the home. In 2021, 62.8% of the sample adult interviews were conducted at least partially by telephone.

### 2.2 Procedures

#### 2.2.1 Outcomes

Participants were asked a series of questions about COVID‐19, including *being told having/tested for COVID‐19* [(1) “Has a doctor or other health professional ever told you that you had or likely had coronavirus or COVID‐19?” (2) “Have you ever been tested for coronavirus or COVID‐19?” (3) “Did the test find that you had coronavirus or COVID‐19?”], *COVID‐19 symptoms* [(1) “How would you describe your coronavirus symptoms when they were at their worst?” “Would you say no symptoms, mild symptoms, moderate symptoms, or severe symptoms?” (2) “When you had coronavirus, did you lose some or all of your sense of smell, or did you smell odors that were not there?” (3) “When you had coronavirus, did you lose some or all of your ability to taste or did you have unwanted tastes or sensations in your mouth that did not go away?”]. Symptoms questions were restricted to only participants who ever had COVID‐19. For *vaccine uptake*, participants were asked [“Have you had a COVID‐19 vaccination?”] and *number of vaccines* [“How many COVID‐19 vaccinations have you received?”]. All answers were yes versus no, except for the severity of symptoms (non/mild vs. medium/severe) and the number of vaccines received (≥2 vs. 1).

#### 2.2.2 Covariates

*Current cigarette smoking* was defined as having smoked at least 100 cigarettes in one's lifetime and now smoking every day or some days. *Current use/smoking of other products* (e‐cigarettes (electronic nicotine delivery systems; ENDS), cigars, pipes/hookah, or smokeless tobacco) was defined as ever using the product and now smoking or using it every day or some days. *Any tobacco product use* was defined as ever used and now using “every day” or “some days” at least one tobacco product. *Any combustible tobacco* product use was defined as using on “every day” or “some days” at least one combustible tobacco product: cigarettes; cigars, cigarillos, filtered little cigars; pipes, or hookahs. Further, we defined exclusive use of each product as currently using only one product (e.g., exclusive ENDS users who did not use any other products).

#### 2.2.3 Independent variables

*Demographic* variables included age groups (18–24, 25–44, 45–64, or ≥65 years), sex (male and female), sexual orientation (straight, lesbian, gay, bisexual, or others), race/ethnicity (non_Hispanic (NH)‐White, NH‐Black, Hispanic, or Other race), education (high school or less, some college, undergraduate, and higher education), poverty level (poor, near poor, and not‐poor), employed (no or yes), social distancing at work in the past 7 days (no or yes), region (household region; Northeast, Midwest, South, and West).

*Disability* was defined based on the self‐reported presence of selected limitations, including vision, hearing, mobility, remembering, self‐care, and communication. Respondents answered six questions: (1) Do you have difficulty seeing, even when wearing glasses? (vision); (2) Do you have difficulty hearing, even when using a hearing aid? (hearing); (3) Do you have any difficulty walking or climbing steps? (mobility); (4) Using your usual language, do you have difficulty communicating, for example, understanding or being understood? (communication); (5) Do you have difficulty remembering or concentrating? (cognition); and (6) Do you have difficulty with self‐care, such as washing all over or dressing? (self‐care). Response items were “no difficulty,” “some difficulty,” “a lot of difficulty,” or “cannot do at all.” Respondents were categorized as having a disability if they reported “a lot of difficulty” or “cannot do” at all to any of the six questions mentioned earlier.^22^, ^23^ These six questions are based on the short set of questions recommended by the Washington Group on Disability Statistics.^22^

*The Kessler Psychological Distress Scale* is a series of six questions that ask about feelings of sadness, nervousness, restlessness, worthlessness, and feeling like everything is an effort in the past 30 days. Participants responded on a Likert scale ranging from “None of the time” (score = 0) to “All of the time” (score = 4). Responses were summed over the six questions; individuals with a score of ≥13 were coded as having serious psychological distress, and respondents with a score of <13 were coded as not having serious psychological distress.^24^

*Chronic health condition was* collected by asking, “Ever been told you had coronary heart disease, high cholesterol, angina, heart attack, stroke, cancer,” or “ever had diabetes, asthma, weak/failing kidneys, weakened immune system due to prescriptions, epilepsy or having a current respiratory allergy.” Those who answered “no” to all questions were considered not to have chronic health conditions.

### 2.3 Data analysis

Unweighted frequencies and weighted percentages with corresponding 95% confidence intervals (CI) of measures associated with COVID‐19 (e.g., ever tested) were calculated overall and by current tobacco product use and other covariates. To assess an association between each COVID‐19 measure with tobacco product use, multivariable models were performed with the COVID‐19 measure (i.e., being told having or tested for COVID‐19, symptoms, testing, and vaccine) as the outcomes and tobacco product use as the exploratory variable controlling for all other study variables (Table 1). Since each COVID‐19‐related measure was considered as an individual outcome, we included those participants who provided information for examined outcome in each regression model. Some independent variables had <1% missingness and were excluded from the analysis. All analyses were weighted using survey procedures in SAS (version 9.4; SAS Institute) to account for the complex survey design and generate nationally representative and unbiased estimates. The statistical significance level was set at *α* = 0.05.

**Table 1:** Demographic characteristics and tobacco products use status by COVID‐19 outcomes among adults in the United States, NHIS, 2021 (*N* = 29,482).

Characteristic	Total sample	Ever had COVID‐19	Ever tested for COVID‐19	Ever had positive test results for COVID‐19	Severity of COVID‐19 symptoms (non/mild vs. medium/severe)	Loss of smell due to COVID‐19	Loss of taste due to COVID‐19	Ever received COVID‐19 vaccine	No. of vaccine (≥2 vs. 1)
Overall	29,482	3494 (13.5)	17,459 (60.8)	3209 (12.5)	2033 (54.8)	2137 (60.5)	2073 (58.2)	16,183 (71.7)	14,293 (87.5)
Sex									
Female	16,102 (51.7)	1949 (14.0)	9770 (62.5)	1799 (13.1)	1178 (56.4)	1255 (62.6)	1220 (60.8)	8907 (72.6)	7914 (88.1)
Male	13,378 (48.3)	1545 (13.0)	7688 (59.0)	1410 (11.9)	855 (53.0)	882 (58.2)	853 (55.1)	7275 (70.7)	6378 (86.9)
Age groups, years									
18–24	1828 (11.4)	305 (17.1)	1227 (66.8)	285 (15.9)	141 (43.4)	198 (59.9)	184 (55.1)	752 (56.9)	612 (81.5)
25–44	9099 (34.1)	680 (16.3)	2966 (67.8)	626 (14.9)	381 (53.5)	491 (68.8)	464 (66.1)	4285 (62.3)	3650 (85.1)
45–64	9592 (32.1)	695 (15.7)	3046 (63.6)	615 (14.4)	406 (57.3)	455 (64.6)	441 (61.5)	5371 (76.0)	4651 (86.8)
≥65	8963 (22.4)	1171 (13.3)	5668 (59.6)	1077 (12.3)	743 (60.7)	700 (60.2)	684 (58.2)	5775 (87.2)	5380 (93.1)
Sexual orientation									
Straight	26,734 (90.3)	3176 (13.5)	15,794 (60.6)	2922 (12.6)	1845 (54.9)	1946 (60.9)	1886 (58.4)	14,891 (71.5)	13,184 (87.8)
Lesbian, gay	602 (2.0)	69 (12.7)	426 (72.9)	61 (12.4)	38 (55.6)	42 (60.0)	43 (64.4)	364 (82.6)	321 (86.5)
Bisexual	576 (2.3)	77 (16.1)	397 (69.6)	67 (13.1)	45 (54.2)	52 (62.0)	50 (62.5)	324 (71.2)	274 (81.1)
Others	1570 (5.5)	172 (14.0)	842 (55.1)	159 (12.0)	105 (53.9)	97 (53.6)	94 (49.7)	604 (69.2)	514 (85.4)
Race/Ethnicity									
NH‐White	19,658 (62.8)	2184 (12.7)	11,277 (59.1)	1949 (11.3)	1294 (56.4)	1288 (58.5)	1274 (57.5)	10,939 (72.9)	9740 (88.2)
NH‐Black	3160 (11.7)	357 (12.7)	2062 (65.8)	352 (12.5)	201 (52.7)	207 (57.5)	211 (56.2)	1586 (65.4)	1377 (86.7)
Hispanic	4081 (16.9)	737 (19.6)	2659 (65.9)	711 (19.2)	431 (54.5)	522 (68.4)	475 (62.1)	2097 (66.3)	1778 (83.9)
Others	2583 (8.6)	216 (9.1)	1461 (56.4)	197 (8.4)	107 (44.7)	120 (53.6)	113 (51.6)	1561 (81.5)	1398 (89.8)
Education									
High school or less	9784 (37.8)	1269 (14.9)	5268 (55.8)	1192 (14.2)	723 (53.3)	785 (61.5)	757 (59.2)	4583 (60.2)	3929 (84.8)
Some college	4453 (15.1)	581 (15.2)	2664 (62.6)	543 (14.3)	368 (57.2)	357 (58.9)	360 (57.5)	2258 (68.8)	1984 (87.0)
Undergraduate	10,660 (33.9)	1237 (12.6)	6537 (63.2)	1116 (11.4)	729 (56.4)	778 (61.9)	746 (58.7)	6286 (78.4)	5586 (88.5)
Higher education	4433 (13.1)	389 (10.0)	2899 (66.9)	340 (8.7)	205 (52.2)	208 (56.0)	199 (53.3)	2981 (90.4)	2727 (90.9)
Poverty level									
Poor	2895 (9.9)	365 (15.2)	1632 (58.2)	338 (14.3)	217 (57.1)	246 (69.1)	223 (62.3)	1218 (54.4)	1011 (83.6)
Near poor	5127 (17.5)	634 (14.4)	2823 (57.3)	594 (13.7)	386 (55.8)	421 (65.7)	403 (60.8)	2398 (60.5)	2051 (84.7)
Not‐poor	21,460 (72.6)	2495 (13.1)	13,004 (62.0)	2277 (12.0)	1430 (54.2)	1470 (57.8)	1447 (56.8)	12,567 (76.6)	11,231 (88.4)
Employed									
No	12,043 (37.8)	1063 (10.3)	6238 (53.0)	971 (9.7)	629 (57.2)	586 (57.6)	582 (55.9)	6955 (74.2)	6264 (88.8)
Yes	16,461 (62.2)	2320 (15.4)	10,710 (65.8)	2134 (14.3)	1337 (54.0)	1486 (61.9)	1427 (59.3)	8957 (70.3)	7800 (86.7)
Social distancing at work									
No	2725 (17.0)	430 (16.9)	1733 (65.5)	390 (15.7)	242 (53.1)	278 (62.2)	264 (58.1)	1588 (64.2)	1415 (89.3)
Yes	13,573 (83.0)	1869 (15.1)	8880 (66.0)	1725 (14.0)	1089 (54.5)	1196 (62.0)	1149 (59.6)	7302 (72.0)	6334 (86.3)
Serious psychological distress									
No	28,461 (96.4)	3349 (13.5)	16,783 (60.6)	3090 (12.6)	1922 (54.0)	2045 (60.4)	1984 (58.0)	15,700 (72.0)	13,880 (87.6)
Yes	1021 (3.6)	145 (13.9)	676 (65.6)	119 (11.9)	111 (75.8)	92 (65.6)	89 (63.0)	483 (61.7)	413 (85.0)
Disability/limitation									
No	26,513 (91.4)	3195 (13.7)	15,800 (61.1)	2937 (12.7)	1824 (53.6)	1979 (61.1)	1909 (58.4)	14,523 (71.6)	12,837 (87.6)
Yes	2967 (8.6)	299 (11.8)	1659 (57.0)	272 (10.9)	209 (70.0)	158 (53.4)	164 (55.0)	1660 (72.7)	1456 (86.6)
Having ≥ 1 health condition									
No	14,512 (54.1)	1838 (14.1)	8649 (61.0)	1690 (13.2)	1003 (50.0)	1183 (61.9)	1106 (58.1)	7319 (66.0)	6322 (85.5)
Yes	14,963 (45.9)	1656 (12.8)	8805 (60.5)	1519 (11.7)	1030 (61.2)	954 (58.7)	967 (58.2)	8861 (78.3)	7969 (89.5)
Region (household region)^a^									
Northeast	4775 (17.5)	498 (12.0)	3000 (64.1)	440 (10.7)	292 (55.2)	296 (57.5)	280 (55.6)	2859 (78.6)	2520 (87.8)
Midwest	6327 (20.8)	793 (14.1)	3570 (58.6)	721 (12.8)	478 (56.3)	479 (59.4)	469 (56.9)	3407 (71.0)	3034 (88.1)
South	10,731 (37.9)	1371 (14.5)	6370 (60.6)	1314 (13.9)	786 (54.6)	857 (62.1)	852 (60.3)	5531 (66.8)	4826 (86.4)
West	7649 (23.8)	832 (12.7)	4519 (60.6)	734 (11.5)	477 (53.6)	505 (60.8)	472 (57.2)	4386 (74.9)	3913 (88.4)

Abbreviations: CI, confidence interval; COVID‐19, coronavirus disease 2019; NH, Non‐Hispanic; NHIS, National Health Interview Survey.

^a^ Northeast: Connecticut, Maine, Massachusetts, New Hampshire, New Jersey, New York, Pennsylvania, Rhode Island, and Vermont; Midwest: Illinois, Indiana, Iowa, Kansas, Michigan, Minnesota, Missouri, Nebraska, North Dakota, Ohio, South Dakota, and Wisconsin; South: Alabama, Arkansas, Delaware, District of Columbia, Florida, Georgia, Kentucky, Louisiana, Maryland, Mississippi, North Carolina, Oklahoma, South Carolina, Tennessee, Texas, Virginia, and West Virginia; West: Alaska, Arizona, California, Colorado, Hawaii, Idaho, Montana, Nevada, New Mexico, Oregon, Utah, Washington, and Wyoming.

## 3 RESULTS

Among US adults in 2021, 18.2% (estimated 46 million) currently used any tobacco product, 14.2% (35.6 million) used any combustible tobacco product, and 3.3% (8.3 million) used two or more tobacco products (Table 2). Overall, 81.8% (207.2 million) of US adults never used tobacco products, 8.7% (22 million) only smoked cigarettes, 3.6% (9.2 million) only used ENDS, 2.9% (7.3 million) only smoked cigars, 0.9% (2.3 million) only smoked pipes, and 2.0% (5.1 million) only used smokeless tobacco. In 2021, 13.5% of US adults (estimated 34.1 million) reported having COVID‐19 (Table 3), 60.8% (153.9 million) ever tested for COVID‐19, 12.5% (31.7 million) ever tested positive for COVID‐19, 54.8% (19.6 million) had medium or severe symptoms (vs. no/mild symptoms) associated with COVID‐19. Among symptomatic adults, 60.5% (21.5 million) and 58.2% (20.7 million) reported a loss of smell or taste, respectively. Overall, 71.7% (133.6 million) had received the COVID‐19 vaccine, of whom 87.5% (116.8 million) received at least two doses.

**Table 2:** Products use status by COVID‐19 outcomes among adults in the United States, NHIS, 2021 (*N* = 29,482).

Characteristic	Total sample	Ever had COVID‐19	Ever tested for COVID‐19	Ever had positive test results for COVID‐19	Severity of COVID‐19 symptoms (non/mild vs. medium/severe)	Loss of smell due to COVID‐19	Loss of taste due to COVID‐19	Ever received COVID‐19 vaccine	No. of vaccine (≥2 vs. 1)
Overall	29,482	3494 (13.5)	17,459 (60.8)	3209 (12.5)	2033 (54.8)	2137 (60.5)	2073 (58.2)	16,183 (71.7)	14,293 (87.5)
Any tobacco products									
No	24,227 (81.8)	2899 (13.8)	14,403 (61.2)	14,403 (61.2)	1686 (54.7)	1745 (59.5)	1699 (57.5)	13,766 (74.9)	12,225 (88.0)
Yes	5255 (18.2)	595 (12.5)	3056 (58.8)	522 (10.9)	347 (55.7)	392 (65.8)	374 (61.7)	2417 (57.3)	2068 (84.9)
Any combustible product									
No	25,274 (85.9)	3084 (14.0)	15,067 (61.4)	2853 (13.1)	1798 (54.8)	1876 (60.2)	1823 (57.9)	14,236 (73.9)	12,626 (87.8)
Yes	4208 (14.1)	410 (10.5)	2392 (57.0)	356 (9.0)	235 (56.2)	261 (63.1)	250 (60.1)	1947 (57.8)	1667 (85.0)
≥2 Tobacco products									
No	28,588 (96.7)	3396 (13.6)	16,916 (60.8)	3126 (12.6)	1987 (55.0)	2067 (60.2)	2015 (58.2)	15,839 (72.5)	14,006 (87.6)
Yes	894 (3.3)	98 (12.3)	543 (60.6)	83 (10.1)	46 (50.0)	70 (71.4)	58 (58.8)	344 (46.7)	287 (85.4)
Tobacco use status									
Nonusers	24,227 (81.8)	2899 (13.8)	14,403 (61.2)	2687 (12.9)	1686 (54.7)	1745 (59.5)	1699 (57.5)	13,766 (74.9)	12,225 (88.0)
Exclusive cigarettes use	2713 (8.7)	242 (9.6)	1439 (52.7)	202 (8.0)	148 (58.7)	148 (61.0)	146 (60.1)	1262 (58.2)	1083 (85.2)
Exclusive ENDS use	926 (3.6)	157 (18.7)	622 (67.9)	138 (16.5)	87 (53.6)	114 (74.0)	103 (65.5)	393 (53.8)	331 (83.7)
Exclusive cigars use	812 (2.9)	85 (10.5)	507 (63.2)	80 (9.8)	44 (52.1)	53 (59.6)	53 (61.7)	415 (64.4)	360 (85.3)
Exclusive pipe/waterpipe use	251 (0.9)	33 (16.4)	180 (71.7)	32 (14.0)	20 (58.0)	24 (73.1)	20 (59.4)	117 (55.3)	101 (85.9)
Exclusive smokeless tobacco use	553 (2.0)	78 (15.1)	308 (56.2)	70 (13.6)	48 (54.8)	53 (63.0)	52 (59.1)	230 (51.3)	193 (84.4)

Abbreviations: COVID‐19, coronavirus disease 2019; ENDS, electronic nicotine delivery systems; NHIS, National Health Interview Survey.

**Table 3:** Tobacco products use and COVID‐19 outcomes among adults in the United States, NHIS, 2021 (*N* = 29,482).

Characteristic	Ever had COVID‐19 (yes vs. no)	Ever tested for COVID‐19 (yes vs. no)	Ever had positive test results for COVID‐19 (yes vs. no)	Severity of COVID‐19 symptoms (non/mild vs. medium/severe)	Loss of smell due to COVID‐19 (yes vs. no)	Loss of taste due to COVID‐19 (yes vs. no)	Ever received COVID‐19 vaccine (yes vs. no)	No. of vaccine (≥2 vs. 1)
Any tobacco products								
No	Reference	Reference	Reference	Reference	Reference	Reference	Reference	Reference
Yes	0.88 (0.77–1.01)	0.91 (0.83–1.01)	**0.81 (0.70–0.93)**	0.93 (0.72–1.19)	**1.36 (1.04–1.77)**	1.23 (0.94–1.59)	**0.56 (0.50–0.63)**	0.84 (0.69–1.02)
Any combustible product								
No	Reference	Reference	Reference	Reference	Reference	Reference	Reference	Reference
Yes	**0.73 (0.62–0.85)**	**0.88 (0.79–0.98)**	**0.66 (0.56–0.77)**	0.90 (0.67–1.21)	1.10 (0.81–1.48)	1.03 (0.76–1.39)	**0.58 (0.51–0.66)**	0.81 (0.66–1.01)
≥2 Tobacco products								
No	Reference	Reference	Reference	Reference	Reference	Reference	Reference	Reference
Yes	0.85 (0.63–1.15)	1.03 (0.84–1.26)	0.74 (0.54–1.01)	0.78 (0.46–1.33)	1.49 (0.79–2.81)	0.89 (0.51–1.57)	**0.49 (0.39–0.62)**	0.92 (0.61– 1.39)
Tobacco use status								
Nonusers	Reference	Reference	Reference	Reference	Reference	Reference	Reference	Reference
Exclusive cigarettes	**0.67 (0.54–0.82)**	**0.73 (0.64–0.84)**	**0.53 (0.47–0.72)**	0.90 (0.61–1.33)	1.01 (0.69–1.49)	1.02 (0.68–1.53)	**0.52 (0.44–0.62)**	0.83 (0.63–1.08)
Exclusive ENDS	1.17 (0.92–1.50)	1.17 (0.95–1.44)	1.11 (0.86–1.43)	0.91 (0.59–1.42)	**1.81 (1.09–3.01)**	1.33 (0.82–2.14)	**0.62 (0.49–0.77)**	0.87 (0.59–1.28)
Exclusive cigars	0.84 (0.63–1.12)	1.12 (0.91–1.38)	0.85 (0.64–1.14)	0.97 (0.57–1.68)	1.27 (0.72–2.26)	1.58 (0.89–2.80)	**0.76 (0.59–0.98)**	0.80 (0.52–1.21)
Exclusive pipe/waterpipe	1.12 (0.67–1.88)	**1.73 (1.11–2.69)**	1.06 (0.64–1.74)	0.94 (0.39–2.28)	1.45 (0.47–4.42)	0.82 (0.31–2.15)	0.74 (0.47–1.16)	0.93 (0.41–2.09)
Exclusive smokeless tobacco	1.07 (0.78–1.46)	0.81 (0.64–1.03)	0.97 (0.69–1.35)	0.97 (0.56–1.68)	1.60 (0.89–2.89)	1.45 (0.83–2.56)	**0.40 (0.31–0.52)**	0.90 (0.56–1.44)

*Note*: Each column (and each tobacco‐use‐related question) is one multivariable model adjusted for age, sex, sexual orientation, race/ethnicity, education, poverty level, employment in the last 7 days, self‐reported social distancing at work, serious psychological distress, disability/limitation, having ≥ 1 health condition, and region (household region). Bold‐faced values indicate *p* < 0.05.

Abbreviations: CI, confidence interval; COVID‐19, coronavirus disease 2019; ENDS, electronic nicotine delivery systems; GED, General Educational Development certificate; NHIS, National Health Interview Survey.

In 2021, of users of any type of tobacco products, 12.5% (5.8 million) were ever told by a physician or health professional that they had COVID‐19, 58.8% (27 million) were ever tested for COVID‐19, and 10.9% (5 million) ever tested positive for COVID‐19. Among tobacco product users who provided information on the COVID‐19 vaccine (34.1 million), 57.3% (19.6 million) ever received the COVID‐19 vaccine, and 84.9% of them (16.6 million) received at least 2 doses of the vaccine.

Among tobacco product users who reported contracting COVID‐19; 65.8% (3.9 million) and 61.7% (3.6 million) reported a loss of smell or taste, respectively.

In multivariable regression models, compared to nonusers of tobacco products, those who used any combustible tobacco product were less likely to report COVID‐19 (adjusted odds ratio = 0.73 [95% CI; 0.62–0.85]), to have ever been tested for COVID‐19 (0.88, 0.79–0.98] or ever tested positive for COVID‐19 (0.66, 0.56–0.77). Those who used two or more tobacco products were less likely to receive the COVID‐19 vaccine (0.49, 0.39–0.62) (all *p*‐values < 0.05). Those who used any type of tobacco products (versus nonusers; 1.36, 1.04–1.77) and ENDS users (versus nonusers; 1.81, 1.09–3.01) were more likely to report loss of smell due to COVID‐19. Compared with never users, current exclusive users of cigarettes (0.52, 0.44–0.62), ENDS (0.62, 0.49–0.77), cigars (0.76, 0.59–0.98), and smokeless tobacco (0.40, 0.31–0.52) were less likely to report ever receiving a COVID‐19 vaccine. Exclusive pipe/waterpipe users were more likely to have been tested for COVID‐19 (1.73, 1.11–2.69).

## 4 DISCUSSION

There is inconsistent evidence on whether tobacco users are at higher risk of acquiring COVID‐19, although they are more likely than nonusers to experience more severe sequelae of the disease if they do acquire COVID‐19.^4^ In the 2021 NHIS, combustible tobacco product users (vs. nonusers) were 27% less likely to report having COVID‐19, 12% less likely to have been ever tested for COVID‐19, and 34% less likely to have ever tested positive for COVID‐19. A previous survey of more than 2.4 million Californian adults,^25^ of whom 44,270 contracted COVID‐19, reported that current smokers (vs. never smokers) were 36% less likely to have COVID‐19 infection. Although these results appear to agree with some of our findings, our study provides additional evidence showing that smokers are also less likely to ever get tested for COVID‐19, which may explain the lower rates of reporting COVID‐19 among this group. While the use of combustible cigarettes, in general, was inversely associated with reporting being infected with COVID‐19, this finding should be viewed with caution. There has been conflicting evidence regarding the impact of smoking on the likelihood of COVID‐19 infections and whether nicotine has a protective effect.^4^ Moreover, even if a level of protection is proven, it is completely outweighed by the substantial health adverse effects attributed to tobacco smoking.

Another interesting finding in this study was the association between hookah smoking and higher odds of getting tested for COVID‐19. Unlike other tobacco products that are typically used solitarily, hookah smoking usually occurs in groups, where smokers often pass the same hose to smoke with friends.^26^ It is possible that because of the sharing culture of hookah smoking, smokers are more concerned regarding the risk of infection,^27^ therefore, were more likely to get tested for COVID‐19. Although it is hard to parse out the pipe from a hookah in NHIS (since they were asked within the same item), this finding has important public health implications while awaiting future studies to explore vaccine hesitancy and acceptance based on the specific type of tobacco product as well as the pattern of use (e.g., intensity and frequency).

Users of any tobacco product exclusive ENDS users‐were more likely than nonusers to report loss of smell due to COVID‐19. These results align with previous studies showing that ENDS users versus nonusers experience a higher frequency of COVID‐19‐related symptoms, including loss of smell.^28^ Knowing that a positive coronavirus infection diagnosis has been strongly correlated with changes in smell and taste,^29^ our findings show that this correlation is more likely among exclusive ENDS users than non‐users. However, some initial evidence suggests that constant flavoring chemical exposure,^30^ particularly at high concentrations from ENDS use, may result in olfactory dysfunctions, which include a diminished sense of smell.^30^, ^31^ Therefore, it is possible that COVID‐19 infection could exacerbate the already diminished smell sensation among ENDS users. Nevertheless, given the study's cross‐sectional design, future longitudinal studies are needed.

In general, COVID‐19 vaccine hesitancy has been reported previously^20^, ^32^, ^33^ and can stem from a combination of factors such as misinformation (or misconception), concerns about side effects and safety, distrust of the healthcare system/governments, and pre‐existing beliefs about vaccines. Tobacco users, in general, hesitate to vaccinate more than nonusers.^34^ Although it was not the case for hookah users as alluded to above, this hesitancy could be explained by health consciousness against tobacco use.^34^ In other words, it is possible that adults who are more likely to seek the COVID‐19 vaccine may also be more likely to refrain from tobacco use to avoid harmful consequences.^20^, ^34^ Providing clear and accurate information about the vaccine through campaigns, public health messaging, and healthcare providers can aid dispel myths and provide accurate information to counteract misinformation and vaccine hesitancy, especially among tobacco users. In addition, targeted tailored health communication messaging to resonate with the values and attitudes of tobacco users, while also providing evidence‐based information about the benefits of vaccination.

These study findings are subject to a few limitations. First, responses were self‐reported and were not validated by biochemical testing, and recall information bias may occur. Nevertheless, self‐reported smoking status correlates highly with serum cotinine levels.^35^ Second, because NHIS is limited to the noninstitutionalized US civilian population, the results are not generalizable to institutionalized populations such as the military. Finally, the NHIS Sample Adult response rate of 50.9% can introduce nonresponse bias. However, all analyses were weighted to avoid this limitation.

While the COVID‐19 infection rate seems to be lower among tobacco users, the loss of smell is more pronounced among this high‐risk group of the population who were also less likely to receive COVID‐19 vaccination. Therefore, efforts to increase the uptake of the COVID‐19 vaccine among tobacco users are warranted. In addition, continued monitoring of tobacco product use amid the COVID‐19 pandemic is crucial to inform public health policies and programs, including cessation interventions, and raising awareness about COVID‐19 and tobacco use.

## AUTHOR CONTRIBUTIONS

**Mohammad Ebrahimi Kalan**: Conceptualization; formal analysis; methodology; resources; supervision; validation; writing—original draft; writing—review & editing. **Rime Jebai**: Conceptualization; data curation; formal analysis; methodology; writing—review & editing. **Wei Li**: Conceptualization; data curation; formal analysis; methodology; validation; writing—review & editing. **Prem Gautam**: Conceptualization; formal analysis; funding acquisition; writing—review & editing. **Seyedeh Yasaman Alemohammad**: Conceptualization; methodology; writing—review & editing. **Zeinab Mortazavizadeh**: Conceptualization; methodology; writing—review & editing. **D. Ward Kenneth**: Conceptualization; methodology; validation; writing—review & editing. **Aditya Chakraborty**: Conceptualization; data curation; formal analysis; methodology; writing—review & editing. **Ghader Dargahi Abbasabad**: Conceptualization; formal analysis; methodology; writing—review & editing. **Raed Behaleh**: Conceptualization; methodology; writing—review & editing. **Zoran Bursac**: Conceptualization; formal analysis; methodology; validation; writing—review & editing. **Ziyad Ben Taleb**: Conceptualization; data curation; methodology; software; validation; writing—original draft; writing—review & editing.

## CONFLICT OF INTEREST STATEMENT

The authors declare no conflict of interest.

## TRANSPARENCY STATEMENT

The lead author Mohammad Ebrahimi Kalan affirms that this manuscript is an honest, accurate, and transparent account of the study being reported; that no important aspects of the study have been omitted; and that any discrepancies from the study as planned (and, if relevant, registered) have been explained.

## Data Availability

The corresponding author had full access to all data in this study and takes responsibility for the integrity and accuracy of the data analysis. The data supporting these findings can be publicly accessed on the US Center for Diseases Control and Prevention at https://www.cdc.gov/nchs/nhis/2021nhis.htm.
